# A new Hendra virus genotype found in Australian flying foxes

**DOI:** 10.1186/s12985-021-01652-7

**Published:** 2021-10-13

**Authors:** Jianning Wang, Danielle E. Anderson, Kim Halpin, Xiao Hong, Honglei Chen, Som Walker, Stacey Valdeter, Brenda van der Heide, Matthew J. Neave, John Bingham, Dwane O’Brien, Debbie Eagles, Lin-Fa Wang, David T. Williams

**Affiliations:** 1 Australian Centre for Disease Preparedness (ACDP), Commonwealth Scientific and Industrial Research Organisation (CSIRO), Geelong, Australia; 2 Programme in Emerging, Infectious Diseases, Duke-NUS Medical School, Singapore, Singapore; 3 SingHealth Duke-NUS Global Health Institute, Singapore, Singapore

**Keywords:** Hendra virus, HeV genotype 2, Henipavirus, Flying fox, Fruit bat, Next-generation sequencing, Zoonosis

## Abstract

**Background:**

Hendra virus (HeV) has caused lethal disease outbreaks in humans and horses in Australia. Flying foxes are the wildlife reservoir from which the virus was first isolated in 1996. Following a heat stress mortality event in Australian flying foxes in 2013, a novel HeV variant was discovered. This study describes the subsequent surveillance of Australian flying foxes for this novel virus over a nine year period using qRT-PCR testing of tissues from flying foxes submitted primarily for Australian bat lyssavirus diagnosis. Genome sequencing and characterisation of the novel HeV variant was also undertaken.

**Methods:**

Spleen and kidney samples harvested from flying fox carcasses were initially screened with two real-time qRT-PCR assays specific for the prototype HeV. Two additional qRT-PCR assays were developed specific for the HeV variant first detected in samples from a flying fox in 2013. Next-generation sequencing and virus isolation was attempted from selected samples to further characterise the new virus.

**Results:**

Since 2013, 98 flying foxes were tested and 11 were positive for the new HeV variant. No samples were positive for the original HeV. Ten of the positive samples were from grey-headed flying foxes (GHFF, *Pteropus poliocephalus*), however this species was over-represented in the opportunistic sampling (83% of bats tested were GHFF). The positive GHFF samples were collected from Victoria and South Australia and one positive Little red flying fox (LRFF, *Pteropus scapulatus*) was collected from Western Australia. Immunohistochemistry confirmed the presence of henipavirus antigen, associated with an inflammatory lesion in cardiac blood vessels of one GHFF. Positive samples were sequenced and the complete genome was obtained from three samples. When compared to published HeV genomes, there was 84% sequence identity at the nucleotide level. Based on phylogenetic analyses, the newly detected HeV belongs to the HeV species but occupies a distinct lineage. We have therefore designated this virus HeV genotype 2 (HeV-g2). Attempts to isolate virus from PCR positive samples have not been successful.

**Conclusions:**

A novel HeV genotype (HeV-g2) has been identified in two flying fox species submitted from three states in Australia, indicating that the level of genetic diversity for HeV is broader than first recognised. Given its high genetic relatedness to HeV, HeV-g2 is a zoonotic pathogen.

**Supplementary Information:**

The online version contains supplementary material available at 10.1186/s12985-021-01652-7.

## Background

Hendra virus (HeV) belongs to the genus *Henipavirus* (family *Paramyxoviridae*, order *Mononegavirales*), which currently includes five species: Cedar virus (CedV), Ghana virus (GhV), Hendra virus (HeV), Mojiang virus (MojV) and Nipah virus (NiV) [1]. Two members of the *Henipavirus* genus, HeV and CedV, have been detected in flying foxes in Australia [2]. HeV is highly pathogenic to horses and humans. Experimental studies have shown that CedV is not pathogenic in the animal models studied (ferrets, guinea pigs and hamsters) [2, 3].

HeV first emerged in Hendra, Australia in 1994 where the outbreak was responsible for the death of a horse trainer, illness in a horse strapper, and the death of 20 horses [4]. Five more human HeV cases have since occurred, of which three have died, and all became infected after close contact with an infected horse [5, 6]. Horses become infected after exposure to excretions from flying foxes, also known as fruit bats, or pteropid bats [7].

Bats comprise the order *Chiroptera*, which is divided into two suborders, the Yangochiroptera (microbats) and the Yinpterochiroptera (megabats) [8]. Pteropid bats belonging to the genus *Pteropus* within the family *Pteropodidae*, suborder Yinpterochiroptera, have been identified as reservoir hosts for several zoonotic viruses from the order *Mononegavirales*, namely HeV, NiV, Menangle virus and Australian bat lyssavirus (ABLV).

HeV was first isolated from the uterine fluid of a pregnant grey headed flying fox (GHFF, *Pteropus poliocephalus*) which aborted twin foetuses after being caught on a barbed-wire fence [9]. Virus was also recovered from pooled foetal liver and lung from the same flying fox, and from neonatal lung of a black flying fox (BFF, *Pteropus alecto*), all collected in Brisbane in September 1996. Since 1996 there have been numerous surveillance studies, most utilising a method of collecting urine underneath flying fox camps and testing by real-time PCR. Most positive results have had high cycle threshold (Ct) values (30+) suggesting low viral loads [10]. Virus isolation attempts from flying foxes are rarely successful and remain a challenge [11]. Sero-surveys of flying foxes have been employed to determine infection prevalence but require the capture and restraint of bats for serum collection, which is a labour-intensive activity with ethical implications. To address these limitations, we have undertaken passive surveillance for the molecular detection of HeV in flying fox specimens submitted to our laboratory for the primary purpose of ABLV testing.

Here, we report the detection and identification of a novel HeV genotype (HeV-g2) from flying foxes in Australia. The virus was first detected from a GHFF in 2013 in Adelaide, South Australia, and has since been detected in two other states. The findings from this study provides critical information about the diversity of henipaviruses in Australia and highlights the importance of enhanced surveillance of henipaviruses and other bat-borne pathogens.

## Methods

### Sample collection and processing

Clinical specimens were collected from flying foxes, submitted to the Australian Centre for Disease Preparedness (ACDP) for the diagnosis of ABLV during 2013 to 2021 from South Australia, Victoria, New South Wales, Queensland, and Western Australia. Tissues were prepared as 10% (w/v) homogenates in Dulbecco's PBS (ph 7.6; Oxoid) containing antibiotics (Sigma-Aldrich) using 1-mm silicon carbide beads (BioSpec Products) in a FastPrep24 tissue homogenizer (MP Biomedical). Samples were clarified by low speed centrifugation (1000×*g, *5 min, 4 °C) and the supernatant used for nucleic acid extraction and virus isolation.

ABLV diagnostic testing was undertaken on brain tissue collected from the flying foxes using the fluorescent antibody test (FAT) and quantitative RT-PCR (qRT-PCR), as previously described [12].

### Cell culture and virus isolation

Vero cells (ATCC CCL-81) and primary kidney cells derived from *P. alecto* (PaKi) [13] were used in this study. Vero cells were cultured at 37 °C in 25 cm^2^ flasks in EMEM (Gibco®, ThermoFisher Scientific) containing 10% foetal calf serum (FCS; Gibco®, ThermoFisher Scientific), supplemented with 1% v/v L-glutamine (Sigma-Aldrich), 10 mM HEPES, 0.25% v/v penicillin–streptomycin (Sigma-Aldrich) and 0.5% v/v amphotericin B (Sigma-Aldrich) and). PaKi cells were cultured in DMEM/F-12 media (Gibco®, ThermoFisher Scientific) with 5% FCS and the same supplements.

For virus isolation, growth media was removed from tissue culture flasks, and cell monolayers were washed once with PBS. For each specimen tested, cells were inoculated with 200 µl of tissue homogenate and incubated for 40 min to allow virus adsorption. Inoculum was then removed, and cell monolayers were washed with PBS, then overlaid with culture media containing supplements and 1% (v/v) FCS. Cells were incubated at 37 °C for 7 days and observed regularly for signs of cytopathic effect by light microscopy. Cells were then frozen, thawed and the cell suspension was centrifuged at 1000×*g* at 4 °C to remove cellular debris. Clarified supernatant (500 µl) was then passaged on a fresh cell monolayer. A total of three passes per sample were performed on each cell line, except one sample, for which Vero cells only were used. Passage 3 samples were tested by real time qRT-PCR to detect the presence of replicating HeV genome.

### Histopathology

Formalin fixed paraffin embedded (FFPE) tissues were available from one HeV-g2 positive bat, a GHFF collected from South Australia in January 2013. These were processed by routine histological methods and sections were stained by haematoxylin and eosin and by HeV immunohistochemistry (IHC) according to methods previously described [14]. For the latter, the primary antibody was an antiserum from a rabbit immunised against recombinant expressed Nipah virus nucleoprotein that cross-reacts with HeV.

### Polymerase chain reaction (PCR)

#### RNA extraction

Fifty microlitre of supernatant from processed clinical specimens or following cell culture passage was used for nucleotide extraction using the MagMax 96 Viral RNA Kit (ThermoFisher Scientific) in a MagMAX Express Magnetic Particle Processor (ThermoFisher Scientific) following manufacturer’s instructions. Extracted RNA was used for PCR testing immediately or stored at −80 °C for further use.

#### Quantitative RT-PCR (qRT-PCR)

Two qRT-PCR assays for detection of HeV matrix (M) gene and nucleocapsid (N) gene [15, 16] were used during initial screening of bat samples. Two additional qRT-PCR assays specific for the M and N genes of HeV-g2 were developed. Primers and probes were designed by using Primer Express 3 (Applied BioSystems). The M gene assay was used for initial testing of samples and the N gene assay was then used as a confirmatory test. Sequences of primers and probes are shown in Table 1. The PCR was performed in 96-well plates in a 25 μL reaction volume containing 5 μL of RNA, 12.5 μL of AgPath One-step RT-PCR buffer (Ambion), 1 μL of 25 × reverse transcriptase, 1.25 μL of 18 μM each primer, 1.25 μL of 5 μM TaqMan probe, and 2.75 μL of nuclease free water. The qRT-PCR assays were performed under the following conditions: 10 min at 45 °C for reverse transcription of RNA, 10 min at 95 °C for inactivation of reverse transcriptase, followed by 45 cycles of 95 °C for 15 s, 60 °C for 45 s using a 7500 Real-time PCR system (Applied Biosystems).

**Table 1:** Primers and probes for qRT-PCR and conventional PCR

**qRT-PCR**	
HeV-g2 M gene	
Forward primer	HeV-g2-M-F: 5’-CTGATCTACGTTACGGCAAACCTT-3’
Reverse primer	HeV-g2-M-R: 5’-GGCCCGCTTCACCATCTCTTAC-3’
Probe	HeV-g2-M-P: 5’-FAM-CAGCATTGAATATTGACCCGCCAGTCA-BHQ1-3’
HeV-g2 N gene	
Forward primer	HeV-g2-N-F: 5’-TGCGACAGATCCCAGTAGTATTAAAT-3’
Reverse primer	HeV-g2-N-R: 5’-GGCAGCTTATTCGGCAAAAG-3’
Probe	HeV-g2-N-P: 5’-FAM-CTCTGGTGACGGAACACAAATGCAAATTTC-BHQ1-3’

Conventional PCR		
Primary RT-PCR		
Primer name	Primer sequences	PCR product
Forward primer	HeV-M-5481F 5’- GCCCGCTTCATCATCTCTT-3’	300 bp
Reverse primer	HeV-M-5871R1: 5’CCACTTTGGTTCCGTCTTTG-3’	
Semi-nested PCR		
Forward primer	HeV-M-5481F 5’- GCCCGCTTCATCATCTCTT-3’	210 bp
Reverse primer	HeV-M-5691R2: 5’-GCAATAGCGTTGTTCCTTCTG-3’	

#### Conventional PCR and Sanger sequencing

A nested PCR for detection of HeV M gene was conducted on extracts of tissue samples positive by qRT-PCR. Primer sequences are listed in Table 1. The RT-PCR was performed using SuperScript III One step RT-PCR System with Platinum DNA Polymerase (Invitrogen) according to manufacturer’s instructions. Briefly, a 25 μl reaction volume consisted of 12.5 μl 2 × reaction buffer, 1 μl of SuperScript III RT/Taq Mix, 0.5 μl of each forward and reverse primer (10 μM), and 5 μl RNA. The PCR reaction was performed in a thermocycler (Eppendorf) under the following conditions: 48 °C for 30 min, 94 °C for 2 min, 40 cycles of 94 °C 15 s, 50 °C for 30 s, and 68 °C for 45 s. Nested PCR was performed using HotStarTaq Plus Master Mix Kit (Qiagen) according to the manufacturer’s instructions. Each PCR reaction consisted of 12.5 μl of 2 × Taq polymerase buffer, 0.5 μl (10 μM) of each forward and reverse primer 10 μM), 2.5 μl of CoralLoad concentrate (10x), 5 μl of the first round RT-PCR product. Thermocycling conditions were: 95 °C for 5 min, and 30 cycles of 95 °C for 15 s, 53 °C for 30 s, and 72 °C for 45 s. PCR products were visualised by gel electrophoresis and products of expected size were purified using QIAQuick gel purification system (Qiagen). Purified PCR products were sequenced using the BigDye terminator v3.1 kit on an Applied Biosystems 3130xl Genetic Analyser (Applied Biosystem). Sequences were analysed using Geneious 11.1 (Geneious).

## Next generation sequencing (NGS) and sequence analysis

### Preparation of Illumina DNA libraries from total RNA

The NGS of initial samples from GHFF from South Australia in 2013 was conducted at Duke-NUS Medical School, Singapore in 2016, and subsequent work with three GHFF from Victoria (bats 2020-01-03; Table 4) was carried out at ACDP, Australia. Two different sequencing library preparation methods were used. At Duke-NUS, Illumina libraries were constructed from total RNA using the NEBNext Ultra Directional RNA Library Prep Kit for Illumina (New England Biolabs) in conjunction with NEBNext Multiplex Oligos for Illumina (New England Biolabs), according to the method described previously [17]. At ACDP, TruSeq RNA library Prep Kit V2 (Illumina) was utilised for DNA library preparation, according to manufacturer’s instructions. The purified libraries were quantified using a Qubit Fluorometer and an Agilent 2100 before enrichment.

### Enrichment of viral library

An approach for viral sequence library enrichment was used as previously described [17]. Targeted HeV-g2 genome enrichment was achieved using custom designed, biotinylated 120-mer xGen Lockdown baits (Integrated DNA Technologies). Biotinylated DNA baits complementary to the consensus sequences of HeV and NiV (Additional file 1: Table S1), plus additional baits specific to HeV-g2 were designed (Additional file 1: Table S2). The DNA baits were designed to tile the henipavirus genome at intervals of approximately 500 nt. The xGen hybridisation and washing kit (Integrated DNA Technologies) were used, and capture workflow was followed according to manufacturer’s instructions. The enriched library was purified using the MinElute PCR Purification Kit (Qiagen) and quantified using a Qubit Fluorometer and an Agilent 2100. The concentration of the final libraries was normalised and pooled in equimolar ratios. The library pool was then loaded into a MiSeq Reagent Kit V2 (2 × 150 cycles; Illumina) and sequenced in a MiSeq platform (Illumina) according to the manufacturer’s instructions.

### NGS data analysis

The NGS sequence data was analysed using CLC Genomic Workbench 20 (Qiagen) using standard parameters. The raw reads were quality-trimmed. De novo assembly was then conducted with the unaligned sequence reads to generate longer sequence contigs. The resultant sequences were analysed using the NCBI nonredundant nucleotide database (BlastN) and protein database (BlastX) (https://blast.ncbi.nlm.nih.gov/Blast).

Gaps in the genome sequence obtained from NGS were resolved with multiple PCRs and Sanger sequencing, using primers designed to flank missing regions (sequences available upon request).

### Phylogenetic analysis

The assembled genomes were phylogenetically analysed with selected reference sequences from GenBank. Multiple sequence alignments for the complete genome, N gene, G gene and L gene were undertaken using MAFFT v.7.301 [18] with the auto option to select the most appropriate alignment parameters. Maximum likelihood trees were then created using IQ-TREE v.2.0.6 [19] with model test enabled to choose the best fitting evolutionary models for each dataset. The trees were visualised using the R package ggtree v.1.14.6 [20].

## Results

### Initial detection and identification of a novel HeV genotype

Spleen and kidney samples from a GHFF from South Australia, submitted in 2013 for ABLV exclusion, were tested by qRT-PCR assays targeting the HeV M and N genes. While the M PCR was negative, HeV N was detected in spleen and kidney samples, with a Ct value of 40.9 and 31.3, respectively. This led us to further investigations using Sanger sequencing to confirm the qRT-PCR results. NGS using two platforms, Roche 454 and Illumina MiSeq, using unbiased methods was attempted but was unsuccessful and no viral sequences were detected (methods available on request).

Nested PCR for HeV M produced a specific amplicon, approximately 300 bp in length, from the kidney sample only. The PCR amplicon was sequenced and BLASTn analysis revealed 89% nucleotide sequence identity to HeV, 77–79% to NiV, 66% to CedV, and 61% to GhV.

The PCR was subsequently used for analysis of positive samples detected by qRT-PCR. Multiple single nucleotide polymorphisms were observed between the different positive samples from different bats. Alignment of the sequences is shown in Additional file 1: Figure S1.

**Fig. 1:**
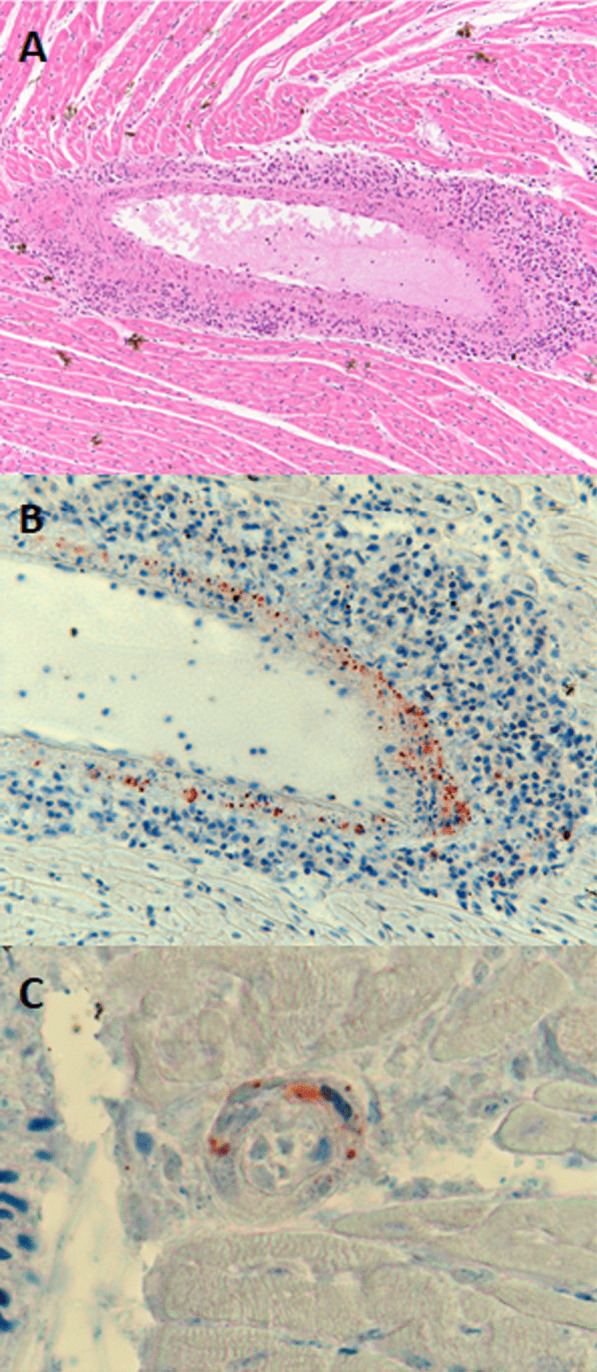
Heart tissue from the grey headed flying fox from South Australia in 2013, showing mononuclear cell inflammation of an artery (**a**, haematoxylin and eosin stain). The walls of this artery (**b**) and a nearby small blood vessel (**c**) contained viral antigen (IHC for henipavirus nucleoprotein)

Histopathology on one HeV-g2 positive GHFF collected from South Australia in January 2013 showed infiltration and cuffing with mononuclear inflammatory cells within the full circumference of one large blood vessel of the heart (Fig. 1a), and IHC showed granular henipavirus antigen present diffusely in the wall of this vessel (Fig. 1b). Antigen was also detected in the wall of one small blood vessel in the same area of myocardium.

Taken together, these results indicated the detection of a novel henipavirus, most closely related to HeV.

### Screening of bat samples using qRT-PCR assay

**Fig. 2:**
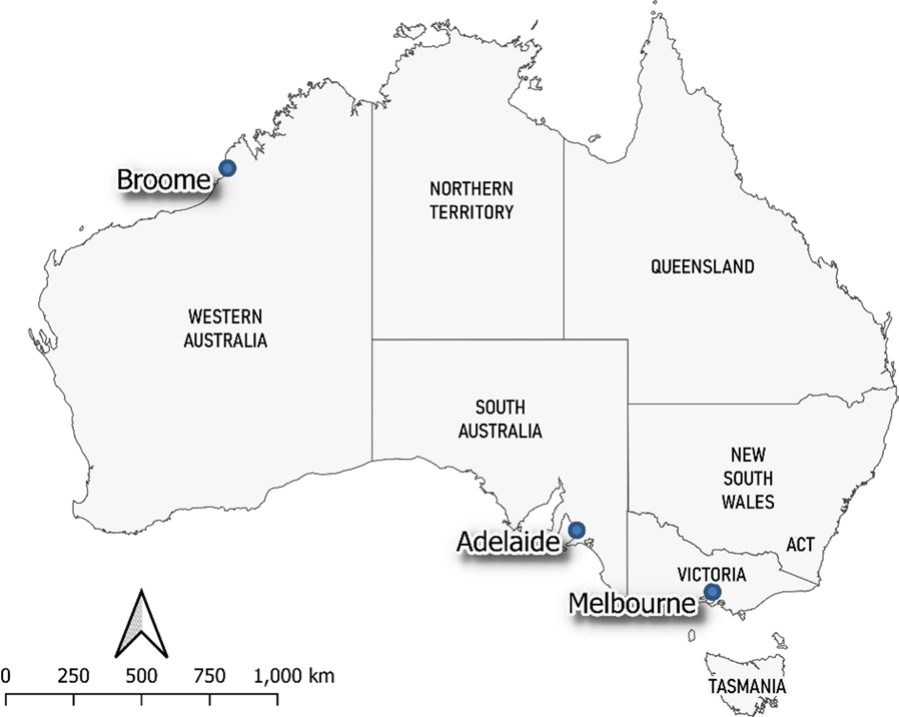
Map of Australia showing the locations of the HeV-g2 positive flying foxes collected between 2013 and 2021: one LRFF was from Broome, three GHFF were from Adelaide and 7 GHFF were from Melbourne. More details on each flying fox can be found in Table 3 and Additional file 1: Table S3

Following the initial detection of HeV-g2 in 2013, a more sensitive qRT-PCR targeting the HeV-g2-M gene was developed and applied to screening bat samples from a broader range of locations in Australia. From January 2013 to April 2021, spleen and kidney samples from 98 flying foxes were tested. The details of species, collection location, and PCR results are summarised in Table 2. More information on each flying fox can be found in Additional file 1: Table S3. Of the 98 bats tested, the majority (n = 81) were GHFF, most of which (n = 77) were from Victoria. Eleven of 98 bats tested positive by qRT-PCR, 10 of which were GHFF, and 1 was a little red flying fox (LRFF) (Table 3; Fig. 2). No samples were positive for prototype HeV. The overall prevalence of HeV-g2 in this study was 11.2%. Of the 11 positive bats, seven were from Victoria, three from South Australia, and one LRFF from Western Australia (Table 3); ten spleen specimens tested positive, whilst only six kidney specimens were positive (Table 4).

**Table 2:** Details of 98 flying foxes tested for HeV-g2 including species, state, year of collection and HeV-g2 results

Species	No. tested	No. positive	State of origin (No.)	Year of collection (No.)
Grey-headed flying fox	81	10	Victoria (71), South Australia (7), NSW (3)	2013 (1), 2014 (11), 2015 (2), 2017 (5), 2018 (9), 2019 (21), 2020 (16), 2021 (16)
Little red flying fox	3	1	Western Australia (3)	2015 (1), 2016 (1), 2017 (1)
Black flying fox	3	0	Western Australia (3)	2014 (1), 2016 (1), 2019 (1)
Unspecified flying fox	11	0	Western Australia (2), Queensland (3), Victoria (6)	2014 (1), 2017 (1), 2018 (1), 2019 (7), 2020 (1)
Total	98	11		

More details on the flying foxes tested in this study can be found in Additional file 1: Table S3

**Table 3:** Details of HeV-g2 positive flying foxes including year and month of collection, species, location, results and cause of death

Year-Sample ID; month	Species	Location	Result spleen	Result kidney	Results confirmed by sequencing?	Cause of death
2013–01 Jan	GHFF	Adelaide, South Australia	Positive	Positive	Yes	Suspect heat stress event
2015–01 Dec	LRFF	Broome, Western Australia	Positive	Negative	No	Dog attack
2019–01 Feb	GHFF	Melbourne, Victoria	Positive	Negative	Yes	Caught in fruit netting
2019–02 Mar	GHFF	Melbourne, Victoria	Positive	Negative	Yes	Caught in fruit Netting
2020–01 Jan	GHFF	Melbourne, Victoria	Positive	Positive	Yes	no history supplied
2020–02 Jan	GHFF	Melbourne,Victoria	Positive	Positive	Yes	Unspecified trauma
2020–03 Jan	GHFF	Melbourne, Victoria	Negative	Positive	Yes	Suspect dog attack
2021–01 Feb	GHFF	Melbourne, Victoria	Positive	Negative	No	Fractured wing
2021–02 Feb	GHFF	Adelaide, South Australia	Positive	Negative	Yes	Dog attack
2021–03 Mar	GHFF	Adelaide, South Australia	Positive	Negative	No	Dog attack
2021–04 Mar	GHFF	Melbourne, Victoria	Negative	Positive	Yes	ABLV*

*GHFF *grey-headed flying fox, *LRFF *little red flying fox

*This bat presented with clinical signs consistent with ABLV infection and tested positive to ABLV byqRT-PCR and FAT

**Table 4:** Comparison of three qRT-PCR assays for the detection of HeV-g2, HeV N assay, HeV-g2 M assay and HeV-g2 N assay using bat samples from 2013 to 2021; values shown are Ct values

Sample ID-sample type	HeV N assay	HeV-g2 M assay	HeV-g2 N assay
2013–01-kidney*^#^	31.3	28.8	NT**
**2013–01-spleen**	40.9	33.4	NT
2015–01-spleen	Negative	38.4	Negative
2019–01-kidney	Negative	Negative	Negative
2019–01-spleen	Negative	34.2	NT
2019–02-kidney	Negative	Negative	Negative
**2019–02-spleen**	Negative	29.6	31.8
**2020–01-kidney**	43.2	30.6	28.3
**2020–01-spleen**^**#**^	34.8	30.6	32.3
**2020–02-kidney**	Neg	34.9	35.5
**2020–02-spleen**^**#**^	34.4	23.1	23.0
**2020–03-kidney**^**#**^	37.1	27.4	26.6
**2020–03-spleen**	Negative	Negative	Negative
2021–01-kidney	Negative	Neg	41.8
2021–01-spleen	Negative	33.2	40.3
2021–02-kidney	Negative	Negative	Negative
**2021–02-spleen**	Negative	37.0	41.3
2021–03-kidney	Negative	Negative	Negative
2021–03-spleen	Negative	36.9	40.2
**2021–04-kidney**	39.0	31.1	31.4
2021–04-spleen	Negative	Negative	36.8
**Total no. of positive**	**7/21 (14%)**	**14/21 (67%)**	**12/18(67%)**

^*^Virus isolation was attempted for samples in bold

^**^NT not tested

^#^NGS was conducted on these samples

### Establishing improved qRT-PCR assays for HeV-g2

In this study, HeV-g2 was detected by three qRT-PCR assays, HeV-N, HeV-g2-M and HeV-g2-N from the flying fox samples. Samples that tested positive using the HeV-g2-M screening assay were then tested by HeV-g2-N gene assay for confirmation. The sensitivity of the three qRT-PCR assays were compared using tissue samples (kidney and spleen) from bats that tested positive. The two HeV-g2 assays developed in this study are more sensitive than HeV-N gene assay for detection of HeV-g2, while both HeV-g2-M and N gene assay have equivalent sensitivity on samples tested. (Table 4). To evaluate the specificity of the newly developed qRT-PCR assays, nine viral RNA samples derived from HeV isolates from equine disease outbreaks in different years were tested. The HeV-g2-M assay detected HeV in all nine samples, while all samples were negative in the HeV-g2-N assay, indicating that the N assay is specific for HeVg2 (Table 5). Two NiV RNA samples extracted from NiV-Malaysia and NiV-Bangladesh isolates tested negative by both HeVg2-M and -N assays, further supporting the specificity of the HeV assays.

**Table 5:** Comparison of qRT-PCR assays for the detection of HeV isolates belonging to the prototype (g1) lineage

Year and Sample ID	HeV-N assay	HeV-g2-M assay	HeV-g2-N assay
2010–01	30.8	29.9	Negative
2015–01	22.7	23.4	Negative
2015–02	21.9	24.8	Negative
2011–01	19.2	22.3	Negative
2011–02	20.3	22.4	Negative
2011–03	20.6	23.3	Negative
2011–04	19.4	18.9	Negative
2011–05	20.7	23.1	Negative
2011–06	30.8	32.4	Negative

### Genomic characterisation by NGS

Spleen or kidney samples that had higher viral loads indicated by qRT-PCR from four GHFF (Table 4.) were selected for hybridization probe enrichment-based NGS analysis, which was not available when NGS was first attempted with the 2013 HeV-g2. The samples generated 1.14–3.59 million paired-end reads per sample with 88–92% of bases ≥ Q30. Following quality trimming of the raw reads, de novo assembly was used to generate long sequence contigs. Blastn analysis demonstrated highest nucleotide identity to HeV genomes, in agreement with the sequences of conventional PCR amplicons. The viral genomes were then assembled based on the HeV genome (GenBank Accession no. AF017149). The gaps in the genome were then filled using multiple PCRs followed by Sanger sequencing.

Using this approach, we have successfully sequenced three full HeV-g2 genomes from three flying foxes from Victoria, as well as a partial genome sequence (approximately 11,530 nt, equivalent to 63% genome) from the flying fox from South Australia originally tested in 2013. The complete genomes are 18,234 nt in length, obeying the ‘Rule-of-Six', which is observed for all known members of the subfamily *Orthoparamyxovirinae* [21] and the genome organisation is identical to HeV. The predicted viral genes and their ORFs are as follows: nucleocapsid protein (N) gene of 1599 nt encoding 532 aa; phosphoprotein (P) gene of 2124 nt encoding 707 aa; matrix protein (M) gene of 1059 nt encoding 352 aa; fusion protein gene of 1641 nt encoding 546 aa; glycoprotein (G) gene of 1812 nt encoding 603 aa; large polymerase (L) protein gene of 6735 nt encoding 2244 aa. The sequences from the four viruses have been submitted to GenBank under Accession Nos: MZ229746-MZ229749.

Comparative analysis of the novel bat HeV full genome with those of other henipaviruses available from GenBank indicated highest nt identity (83.6%) to HeV (AF017149), followed by 71% nt identity to NiV Malaysia and Bangladesh strains (AJ627196 and AY988601), respectively, 57% to CedV (NC025351), 55% to GhV (HQ660219), and 55% to MojV (NC025352). The amino acid sequences of six individual proteins are highly homologous to those of HeV (AF017149), with sequence identities of 96% for N protein, 82% for P protein, 96% M protein, 95% F protein, 92% for G protein, and 95% for L protein. Comparison of nucleotide and protein sequences of each of the six individual genes is demonstrated in Table 6. In addition, there are minor sequence variations among the novel bat HeV genomes from this study, though they share over 99% nucleotide identity at genome level. HeV-g2/2020-01 and -03 are nearly identical with only 5nt differences and one aa change. HeV-g2/2020-02 is slightly diverse from the other two, with 75 nt changes across the genome, and 13 aa changes in different coding regions (Table 7). Analysis of partial genome sequences from HeV-g2/2013-01 from South Australia revealed minor variations in comparison with the 3 viruses from Victoria (data not shown).

**Table 6:** Comparison of sequences of HeV-g2 with other henipaviruses (figures are % similarity)

Virus	Nucleocapsid (N)		Phosphatase (P)		Matrix (M)		Fusion (F)		Glycoprotein (G)		Polymerase (L)	
	nt^2^	aa^3^	nt	aa	nt	aa	nt	aa	nt	aa	nt	aa
HeV (g1)^1^	87	96	83	82	88	96	87	95	85	92	87	95
NiV-M	77	91	70	64	77	90	75	87	71	79	75	87
NiV-B	77	91	71	64	77	90	74	86	71	79	75	87
CedV	62	60	53	27	62	61	54	42	54	30	58	52
GhV	60	56	54	28	62	62	57	52	54	29	59	53
MojV	56	48	53	21	61	60	53	40	51	21	58	52

^1^HeV: Hendra virus (AF017149); NiV-M: Nipah virus Malaysian strain (AJ627196); NiV-B, Nipah virus Bangladesh strain (AY988601)); CedV: Cedar virus (NC_025351), GhV: Ghana virus (HQ660129); MojV: Mojiang virus (NC_025352)

^2^nucleotide sequence identities (%) against cognate nucleotides of HeV-g2 virus

^3^amino acid sequence identities (%) against cognate proteins of HeV-g2 virus

**Table 7:** Comparison of nucleotide and amino acid sequence variations of HeV-g2 viruses when compared to the consensus sequence for HeV-g2

Virus	Full genome (%)	Open reading frame length (nt)					
		N 1599 nt aa	P 2124 nt aa	M 1059 nt aa	F 1641 nt aa	G 1812 nt aa	L 6735 nt aa
HeV/Australia/*Pteropus poliocephalus*/2020–01	5* (0.02%)^#^	1 0	1 0	0 0	1 0	1 1	0 0
HeV/Australia/*Pteropus poliocephalus*/2020–02	75 (0.4%)	7 0	9 3	4 0	11 1	6 2	24 7
HeV/Australia/*Pteropus poliocephalus*/2020–03	5 (0.03%)	1 0	1 0	0 0	0 0	0 0	3 0

^*^Total number of nt and aa changes across the full genome, including NCRs

^#^Percentage variation across the full genome, including NCRs, compared to consensus sequence

### Phylogenetic analysis

**Fig. 3:**
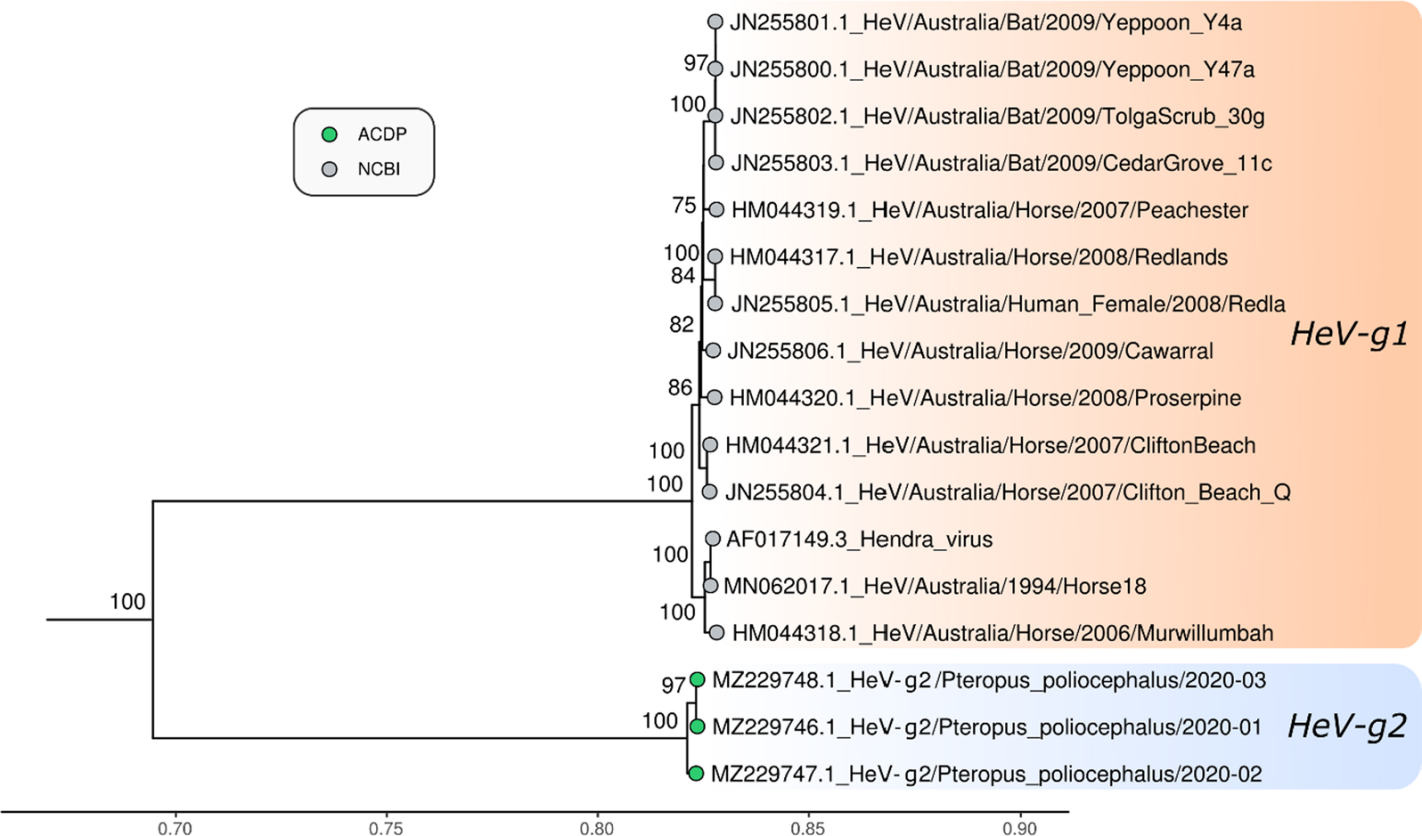
Maximum likelihood phylogenetic tree of complete Hendra virus genomes available in GenBank. The TN model with gamma rate heterogeneity was used as the best fit by IQ-TREE v.2.0.6. The results from 1000 bootstrap replicates are given on the nodes (if greater than 70) and the scale represents the number of nucleotide substitutions per site. The tree was drawn with Nipah virus outgroups which were removed for visualisation

**Fig. 4:**
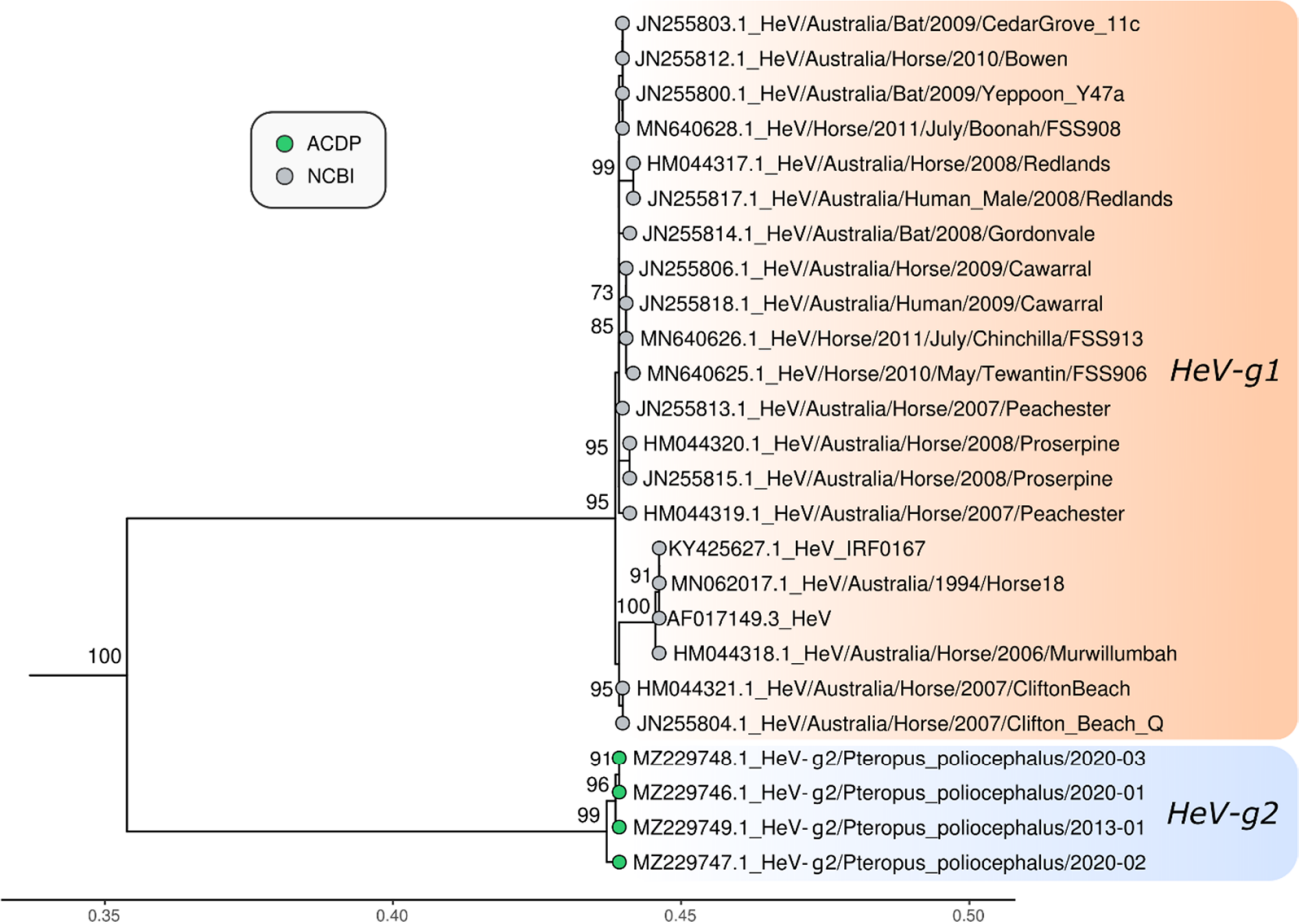
Maximum likelihood phylogenetic tree of N gene nucleotide sequences. The TIM2 model allowing for a proportion of invariant sites was used as the best fit by IQ-TREE v.2.0.6. The results from 1000 bootstrap replicates are given on the nodes (if greater than 70) and the scale represents the number of nucleotide substitutions per site. The tree was drawn with Nipah virus outgroups which were removed for visualisation

**Fig. 5:**
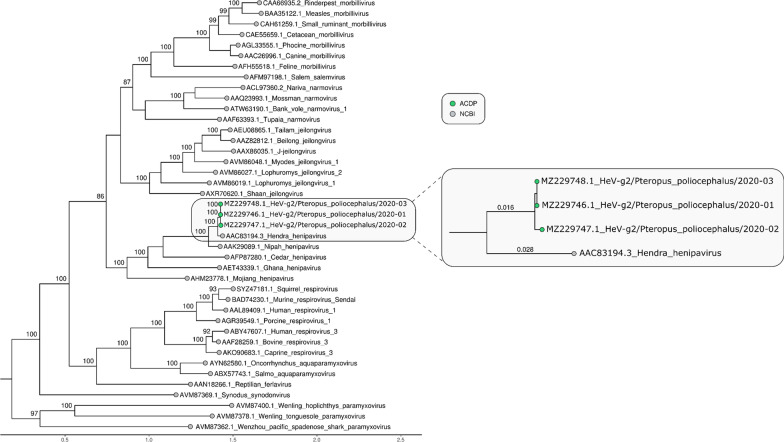
Maximum likelihood phylogenetic tree of L gene amino acid sequences. The tree was created using the JTT substitution model in IQ-Tree v.2.0.6 as per ICTV guidelines for the family *Paramyxoviridae* (Rima et al., 2019). The results from 100 bootstrap replicates are given on the nodes (if greater than 70) and the scale represents the number of substitutions per site. The zoomed inset has lengths written on the branches specifically showing the distance between HeV-g1 and HeV-g2

Maximum likelihood phylogenetic analysis based on the full genome, nucleocapsid (N), glycoprotein (G) and polymerase (L) of the three novel genomes sequenced in this study and reference genomes of other members of the genus *Henipavirus*, and subfamily *Orthoparamyxovirinae* was undertaken. The three sequences clustered together with HeV, but as a separate sub-lineage in the phylogenetic trees of full genome, N and L protein, with 100% bootstrap support for the two HeV sub-lineages (Figs. 3, 4, 5). Analysis of a partial region of the G gene, including the HeV-g2/2013-01 virus revealed a very similar tree topology to the full genome and N gene phylogenetic trees (Additional file 1: Figure S2). Based on the ICTV species demarcation criteria, a new species in the genus *Henipavirus* is defined as having a distance greater than 0.03 between the tip of the branch and the nearest node to the reference sequence (GenBank Accession No. AAC83194.3) in a phylogenetic tree of the complete L protein [1]. Phylogenetic analysis of the complete L protein of the novel HeV detected in this study indicate that these viruses are HeV species (branch length to the nearest node is < 0.03). As these viruses form a distinct sub-lineage, we propose to designate this group as HeV genotype 2 (HeV-g2), and designate the previously identified sub-lineage, containing the reference HeV sequence, as genotype 1 (HeV-g1). Therefore, the strain names of the 3 viruses with full genome from 2020, and one virus with partial genome from 2013 are: HeV-g2/Australia/*Pteropus poliocephalus*/2020–01, HeV-g2/Australia/*Pteropus poliocephalus* /2020–02, HeV-g2/Australia/*Pteropus poliocephalus* /2020–03, and HeV-g2/Australia/*Pteropus poliocephalus* /2013–01.

### ABLV testing

All but three samples tested negative for ABLV. One positive sample (2021–04) was from a GHFF, collected in Melbourne, 2021. It had presented with clinical signs consistent with ABLV infection. It also tested positive to HeV-g2. The two other ABLV positive bats did not test positive for HeV-g2. They were both GHFF; one collected in Adelaide in 2021 and one collected in Melbourne, 2018 (Additional file 1: Table S3).

## Discussion

A new genotype of HeV has been identified in flying foxes in Australia. This follows the first detection of HeV-g1 in Australian flying foxes in 1996 [9], and Cedar virus in 2012 [2]. Histopathology, including IHC, indicated that this virus replicates within blood vessels and is able to cause significant vascular disease in one flying fox. However it is unknown if there were any associated clinical manifestations of disease in this animal that died from an unrelated suspected heat stress event.

Molecular characterisation of the HeV-g2 demonstrated highest genetic similarity to HeV-g1, when compared to other members of the genus. Sequence variation was observed within HeV-g2 despite high sequence similarity (> 99% at genome level) between each other. This is similar to the findings from HeV (g1) in flying foxes and horses [22, 23]. The high level of genetic similarity between HeV-g1 and HeV-g2 indicates that the latter shares the pathogenic phenotype of the prototype HeV-g1 and can also be considered as a zoonotic pathogen. To date, there has been no report of spill-over events of HeV-g2 in the areas where the virus has been detected in flying foxes. However, the recent retrospective identification of a virus belonging to this sub-lineage from a 2015 case of equine neurological disease in Queensland shows the HeV-g2 is able to infect horses and potentially cause disease [24]. Further studies on epidemiology and pathogenicity of HeV-g2 are warranted.

In this study, 98 bats were tested by PCR and HeV-g2 was detected in 11, with a detection rate of 11.2%. In a similar study where archived tissues from 295 flying foxes collected from Queensland between 1996 and 1997 were screened, HeV-g1 was detected in 20 individual flying-foxes (6.4%) from various tissues; detection was significantly higher in BFF and the spectacled flying-fox (SFF), with 10 (22.7%) and 9 (8.6%) detections respectively. There was a single detection in GHFF (1%), and no detections in LRFF (n = 50) [10]. As was observed in our study, there were more detections in spleen than kidney, suggesting that spleen might be the tissue of choice in future studies.

Ten of the eleven bats that tested positive for HeV-g2 were GHFF (Table 3). In a previous study [25], 1410 individual BFF were tested by qRT-PCR, and HeV-g1 was detected in 43 animals with 3% prevalence of detection. There were no positives in the 1168 GHFF and 262 LRFF sampled. Edson’s study sampled urine, serum, packed haemocytes and a variety of swabs collected from free-flying and presumably healthy populations. The difference in prevalence between this study and ours is most likely due to the sampling bias in our study which used tissue samples from compromised flying foxes.

It is unclear whether infection of HeV-g2 occurs in BFF, and what level of prevalence exists, as only very limited numbers (n = 3) of BFF were tested in this study. It is interesting that one of three LRFF from Western Australia tested positive for HeV-g2. However, this was at the limit of detection and could not be confirmed by sequencing. Additional surveillance in these populations is needed.

Hendra and Nipah viruses are serologically cross-reactive [14, 26]. Although serological evidence of HeV infection has been found in all four flying fox species in Australia, including the SFF, BFF, LRFF and GHFF [27], the discovery of HeV-g2 raises the question of which variant the flying foxes have been exposed to—HeV-g1, HeV-g2 and/or other variants. Current serological tests would not be able to differentiate between the different variants.

Epidemiological studies have suggested that only the BFF and SFF are the primary reservoir hosts for HeV [10, 25, 28, 29]. However all of these studies used an assay which only detected HeV-g1 [15]. Another study showed that the density of BFF and SFF had a strong positive correlation with equine Hendra case locations, also implicating these species as the primary reservoir in Hendra virus infection of horses [30]. Neither GHFF nor LRFF have been identified as the principal source of HeV in spill-over events to horses, despite reports of high levels of seroprevalence to HeV in these species, and the isolation of virus from GHFF [9, 31, 32]. It is notable that in this study HeV-g2 appears to have a broad geographic distribution, in areas of Australia that were previously deemed as low risk for HeV spill-over events.

Co-infection with HeV-g2 and ABLV was detected in one GHFF. This is the first time that this has been reported. Virus co-infection in flying foxes has not been studied extensively but is known to occur. While mean viral prevalence was low, multi-viral shedding from flying fox populations was common in one report, with up to eight paramyxoviruses, which included HeV-g1, detected in pooled urine samples from one mixed colony (containing BFF and GHFF) at a single point in time, and referred to as a ‘synchronous shedding pulse’ [33].

A qRT-PCR assay targeting the HeV N gene was used during initial detection of the HeV-g2 from GHFF from South Australia in 2013, in addition to the HeV-g1-specific M gene assay that failed to detect HeV-g2. This demonstrates the benefit of using diagnostic qRT-PCR tests that target multiple genes for disease investigations to address circulating genetic variants. Subsequently, a new qRT-PCR assay based on the HeV-g2 M gene was developed and applied to the screening of samples from flying foxes. This assay is more sensitive than the HeV N gene assay for detection of HeV-g2, and it is also able to detect HeV belonging to the prototype lineage. The broad reactivity of this assay could potentially be beneficial to the detection of mutant/variant forms of HeV in the future. Through obtaining more sequences of HeV-g2 from GHFFs during this study, we developed an additional HeV-g2 specific qRT-PCR assay targeting the N gene. This assay is specific for HeV-g2 and is used as the confirmatory test in our laboratory. Preliminary validation of both assays using bat samples submitted for disease exclusion, equine samples from previous outbreaks and NiV isolates indicated that these tests perform with high levels of sensitivity and specificity. Further assessment of these tests will be required for full validation according to World Organisation for Animal Health principles for veterinary diagnostic tests [34].

As it has been shown that HeV-g2 can spill over from flying foxes to another species, namely the horse, this novel virus threatens animal health and potentially human health. These new assays enhance diagnostic capability through rapid and specific detection of HeV-g2. The addition of capability for detection of HeV-g2 to current laboratory diagnostic testing algorithms for the diagnosis of HeV infections in animals of different species, particularly horses, is of critical importance. An updated testing protocol to incorporate these tests has been applied at ACDP.

In the present study, virus isolation was attempted using the continuous Vero cell line and primary *Pteropus alecto* (kidney) cell culture. HeV-g2 was unable to be isolated from selected PCR positive GHFF samples. Previously, HeV has been isolated from flying fox urine using these cell cultures, with equal success in either Vero cells or primary bat cell lines [23]. The unsuccessful attempts for isolation of HeV-g2 virus may be due to non-viability of the virus in the samples due to poor sample quality. These samples were collected either from carcasses exposed to ambient temperature for unknown periods, or euthanised and stored under suboptimal conditions for the preservation of live virus. The low virus load in most of the samples, as indicated by qRT-PCR results, may have also been a contributing factor.

## Conclusions

The identification and characterisation of a novel HeV genotype, designated HeV-g2, in two species of flying fox previously deemed to be low risk for HeV cross-species transmission, in regions where HeV spill-over has not occurred, is a significant contribution to HeV epidemiology in Australia. The new variant causes vasculitis in the flying fox host and it is has been associated with a fatal illness in a horse. Due to high levels of genome similarity with the HeV prototype lineage, HeV-g2 can be considered a zoonotic pathogen, posing a significant risk to different species of animals, particularly horses and humans. Further in-depth studies on the epidemiology, pathogenicity, and risk of spill-over of this new virus are warranted. Development of the new RT-qPCR assays reported in this study will significantly improve the detection, diagnosis and surveillance of HeV-g2 in flying foxes and other host species.

## Supplementary Information

**Additional file 1**.** Table S1**: Hybridization probes designed based on HeV and NiV genome sequences.** Table S2**: Hybridization probes set-2 which is a combination of set-1 and HeV-G2 sequences.** Table S3**: Details of all flying foxes tested in this study, including year and month of collection, species, location, ABLV and HeV-g2 results (positive results are highlighted in grey) and the suspected cause of death.** Figure S1**: Alignment of HeV-g2 M gene sequences amplified by PCR.** Figure S2**: Maximum likelihood phylogenetic tree of 568 bp of the glycoprotein G gene nucleotide sequences.

## Data Availability

All data generated or analysed during this study are included in this published article and supplementary information files. The genome sequences of the three HeV-g2 are available at the GenBank (https://www.ncbi.nlm.nih.gov/genbank/).

## Abbreviations

aa: Amino acid
ACDP: Australian Centre for Disease Preparedness
HeV: Hendra virus
HeV-g2: Hendra virus genotype 2
ABLV: Australian bat lyssavirus
GHFF: Grey-headed flying fox
qRT-PCR: Quantitative reverse transcription polymerase chain reaction
NGS: Next-Generation Sequencing
nt: Nucleotide
