# Supplementary material for: A new Hendra virus genotype found in Australian flying foxes
Source: Virol J. 2021 Oct 13;18:197. doi: 10.1186/s12985-021-01652-7 (PMC8510678; doi:10.1186/s12985-021-01652-7)
Supplement: Supplementary file 1 — Additional file 1. Table S1: Hybridization probes designed based on HeV and NiV genome sequences. Table S2: Hybridization probes set-2 which is a combination of set-1 and HeV-G2 sequences. Table S3: Details of all flying foxes tested in this study, including year and month of collection, species, location, ABLV and HeV-g2 results (positive results are highlighted in grey) and the suspected cause of death. Figure S1: Alignment of HeV-g2 M gene sequences amplified by PCR. Figure S2: Maximum likelihood phylogenetic tree of 568 bp of the glycoprotein G gene nucleotide sequences. [file 12985_2021_1652_MOESM1_ESM.docx]

**Supplementary Figure S1.** Alignment of HeV-g2 M gene sequences amplified by PCR

Consensus    GCCCCCTTCACCATCTCTTACCATGGAAAAAAGTTTTGACTGGCGGGTCAATATTCAATG  60

2013-01     ............................................................

2019-01     ............................................................

2019-02      ......................G.....................................

2020-01      ............................................................

2020-02      ............................................................

2020-03      ............................................................

Consensus    CTGTCAAGGTTTGCCGTAACGTAGATCAGATTCAACTGGAAAAGCAACAATCATTGAGGA  120

2013-01      ............................................................

2019-01      ............................................................

2019-02     ............................................................

2020-01     ............................................................

2020-02      ............................................................

2020-03      ............................................................

Consensus    TTTTTTTCCTAAGTATTACCAAATTGAATGATTCCGGTATCTACATGATCCCGAGAACAA  180

2013-01     ....................................................A.......

2019-01     ....................................................A.......

2019-02      ............................................................

2020-01      ............................................................

2020-02      ............................................................

2020-03      ............................................................

Consensus    TGTTGGAATTCAGAAGGAACAATGCCATTGCTTTTAATCTTCTGATATACCTCAAAATTG  240

2020-02      ............................................................

2019-02      ............................................................

2013-01     ............................................................

2019-01     ............................................................

2020-01      ............................................................

2020-03      ............................................................

Consensus    ATGCAGATCTTGCAAAAGCCGGAATCCAAGGGAGTCTCGACAAGGACGGAGCTAAAGTGG 300

2013-01      ............................................................

2019-01      ............................................................

2019-02      ............................................................

2020-01     ............................................................

2020-02      ..................................C.........................

2020-03     ..................................C.........................


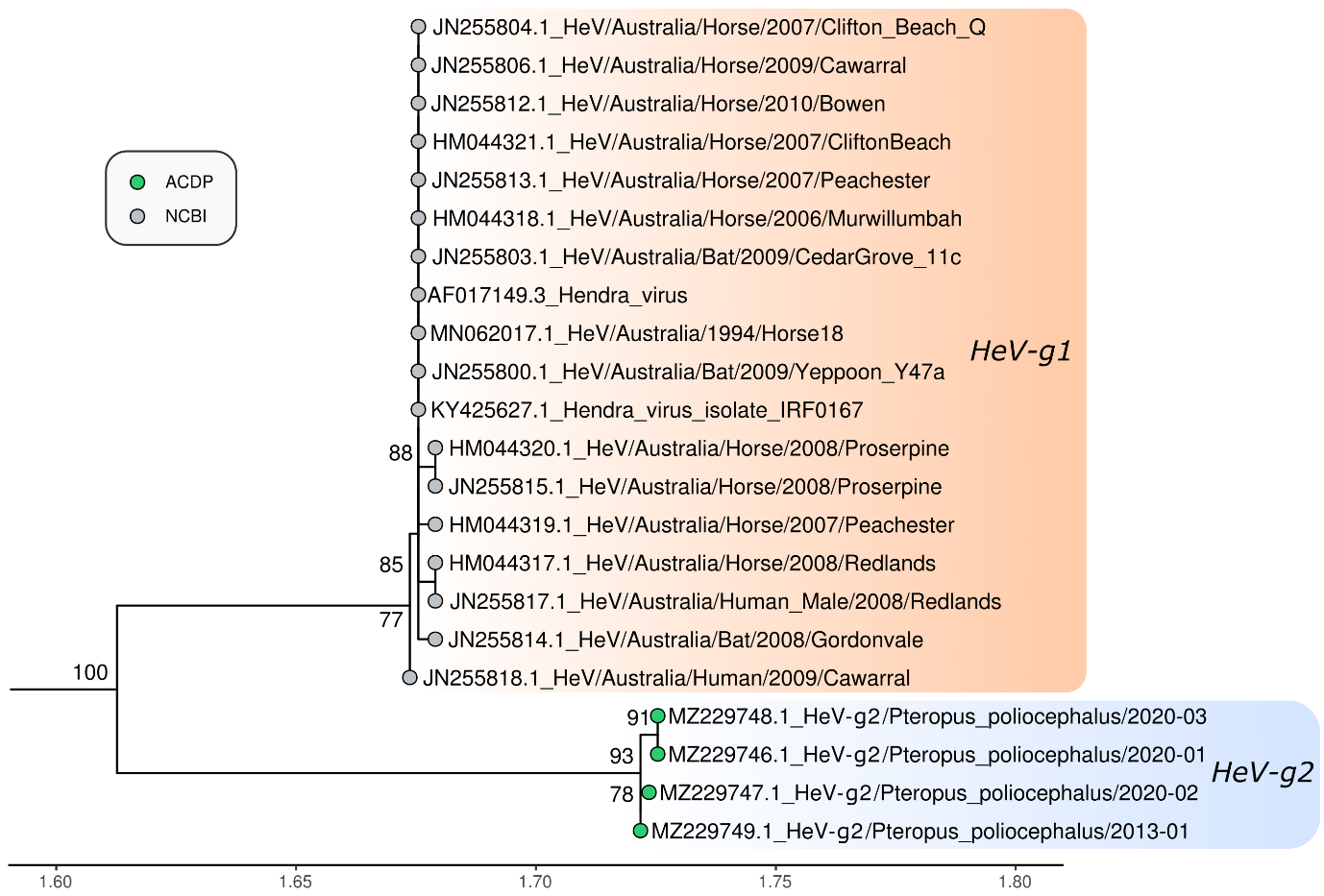


Supplementary Figure S2. Maximum likelihood phylogenetic tree of 568 bp of the glycoprotein G gene (recovered in all samples). The HKY model with empirical base frequencies and allowing for a proportion of invariant sites was used as the best fit by IQ-TREE v.2.0.6. The results from 1000 bootstrap replicates are given on the nodes (if greater than 70) and the scale represents the number of nucleotide substitutions per site. The tree was drawn with Nipah virus outgroups which were removed for visualisation.

Table S1. Hybridization probes designed based on HeV and NiV genome sequences

| NiV bait-01 | ATGAGTGATATTTTTGAAGAGGCGGCTAGTTTTAGGAGTTATCAATCTAAGTTAGGAAGAGATGGGAGGGCTAGTGCGGCAACTGCTACTTTGACAACCAAGATAAGGATATTTGTACCG |
| --- | --- |
| NiV bait-02 | GAGTCTATCAGTAAGGAAGTTCATGGTTGAGATCCTCATAGAAGTCAAGAAAGGAGGATCTGCTAAAGGCAGAGCAGTAGAAATAATCTCAGACATCGGAAACTATGTCGAGGAAACTGG |
| NiV bait-03 | GCAGAACTCGCTGCTGCAGTTCAGGAAACATCAGCAGGAAGGCAAGAGAGTAATGTTCAGGCTAGAGAGGCAAAATTTGCTGCAGGAGGTGTGCTCATTGGAGGCAGTGATCAAGATATC |
| NiV bait-04 | GGGATCAACCATATCAAAGTTACGAATCGTTTTAATTATATTAATCAAATGATACTCTTTTATGGGCAAACCGAAGAACCAATGTCTACATGTAAATTGAGCTTTGGTATTGCAATCTAA |
| NiV bait-05 | CATTCAATGGATAAATTGGAACTAGTTAATGATGGCCTCAATATTATTGACTTTATTCAGAAGAACCAAAAAGAAATACAGAAGACATACGGACGATCAAGCATTCAACAACCCAGCATC |
| NiV bait-06 | GGGAATGTATGTCTTGTATCTGATGCAAAGATGCTGTCCTATGCTCCCGAAATTGCAGTTTCTAAAGAAGATCGGGAAACTGATCTAGTTCATCTTGAGAATAAACTATCTACTACAGGA |
| NiV bait-07 | CCAGGAGCAGGACCGAAAGATTCAGCAGTGAAGGAAGAACCACCCCAGAAGAGGCTACCTATGTTAGCTGAAGAATTTGAGTGCTCTGGATCGGAAGACCCAATCATTCGGGAGCTGCTG |
| NiV bait-08 | GACAACGACTCACTTGATGATAAATATATCATGCCTTCAGATGATTTCTCAAACACTTTCTTCCCGCATGACACTGATCGCTTAAATTATCACGCAGACCACTTAGGTGATTATGACCTC |
| NiV bait-09 | AGATCTTATTCTTCCTGAACTTAATTTTGAGGAGACAAATGCATCTCAATTTGTTCCTATGGCAGATGATTCATCCAGAGATGTTATCAAGACATTGATAAGGACTCACATTAAAGATAG |
| NiV bait-10 | GAAGGAGTATCTGATTTCAGCCCTAGTTCTTGGGAGCATGGTGGGTATCTTGATAAGGTTGAACCAGAAATTGATGAAAATGGCAGTATGATTCCAAAATACAAGATCTATACCCCAGGA |
| NiV bait-11 | GATTCTGGAATCTACATGATTCCACGAACCATGCTTGAGTTCAGGAGAAACAATGCCATTGCCTTCAATCTTCTAGTGTACTTGAAGATTGATGCTGATTTATCCAAAATGGGGATCCAG |
| NiV bait-12 | TGGTAGTTATACTTGACAAGAGATGTTATTGTAATCTTTTAATATTGATTTTGATGATCTCGGAGTGTAGTGTTGGGATTCTACATTATGAGAAATTGAGTAAAATTGGACTTGTCAAAG |
| NiV bait-13 | TGGCCGGAGTTATAATGGCAGGAGTTGCTATTGGAATTGCAACCGCAGCTCAAATTACTGCAGGTGTAGCATTATATGAGGCAATGAAAAATGCTGACAACATCAACAAACTCAAAAGCA |
| NiV bait-14 | ATGGATCAGCATTGTCCCAAATTTCATATTGGTAAGGAACACATTAATATCAAATATAGAGATTGGATTTTGCCTAATTACAAAGAGGAGTGTGATCTGCAACCAAGATTATGCAACACC |
| NiV bait-15 | TCCAGCATGAATCAGTCCTTACAACAGTCTAAGGACTATATCAAAGAGGCTCAACGACTCCTTGATACTGTTAATCCATCATTAATAAGCATGTTGTCTATGATCATACTGTATGTATTA |
| NiV bait-16 | CATGGACATTAAGAAAATAAATGAAGGATTATTGGACAGCAAAATATTAAGTGCTTTCAACACAGTAATAGCATTGCTTGGATCTATCGTGATCATAGTGATGAATATAATGATCATCCA |
| NiV bait-17 | CCAGCTAATATTGGGCTGTTAGGTTCAAAGATCAGCCAGTCAACTGCAAGTATAAATGAGAATGTGAATGAAAAATGCAAATTTACACTGCCTCCCTTGAAAATCCACGAATGTAACATT |
| NiV bait-18 | GTATGATAAAGTTATGCCGTATGGACCTTCAGGCATCAAACAGGGTGACACCCTGTATTTTCCTGCTGTAGGATTTTTGGTCAGGACAGAGTTTAAATACAATGATTCAAATTGTCCCAT |
| NiV bait-19 | GCATTCCTAATTGACAGAATCAATTGGATAAGCGCGGGTGTATTCCTTGACAGCAATCAGACCGCAGAAAATCCTGTTTTTACTGTATTCAAAGATAATGAAATACTTTATAGGGCACAA |
| NiV bait-20 | TCTTGATCGGTGATCTTTGAGAACAATGATATCATATAGTTCATCAAGTGATAATCAATTCTTTATATGTACACTTTAGAGTATATTTTGAGACTTAGTATTTTCGGCCCGAATGTTAAA |
| NiV bait-21 | AGATGGCCGATGAATTATCCATATCTGACATCATCTACCCTGAATGTCATTTGGATAGTCCTATAGTCTCTGGTAAACTAATATCAGCTATTGAATATGCTCAATTGAAACACAATCAAC |
| NiV bait-22 | TTTTGTTTTGGTTTACTATCAAAACAGAGATGCGAGCAGTGATCAAGAATTCGCAAAAGCCGAAATTCCGTTCAGATTCATGCATAATACACATGCGAGACAAAAGTACTGAAATAATCC |
| NiV bait-23 | TATCGATAATATACATCTGTTGGCAGAGTTCTTTTCTTTCTTTCGTACGTTTGGCCATCCTATTCTTGAGGCTAAAGTTGCTGCAGAAAAAGTGAGAGAACATATGTTGGCAGATAAAGT |
| NiV bait-24 | TCAGGTGTCGGTAAATATTTTAAGGAGAACGGGATGGTTAAGGATGAGCACGAACTTTTGAAGACACTCTTCCAATTGTCTATTTCCTCAGTTCCTCGAGGGAACAGTCAGGGTAATGAT |
| NiV bait-25 | CATGGACTATAGCAACTATCCCCTTTTTATTCTTGAGTGCCTATGAGACAAACACGAGGATTGCTGCAATTGTCCAAGGAGACAATGAATCAATTGCTATCACTCAAAAAGTTCATCCTA |
| NiV bait-26 | ATTACCTTAATTTGTCTAGAATTTTTGTTAGGAATATAGGTGATCCGGTCACAGCATCTTTGGCTGATCTCAAAAGAATGATTGACCACAGCATTATGACTGAAAGCGTATTACAAAAAG |
| NiV bait-27 | CTTGAGGCTATGGTGGGAAGGTATATAACAGGGAGCTTAGAGTGCCAAATTTGTGAGCAGGGAAACACGATGTATGGGTGGTTCTTTGTACCTAGGGATTCCCAATTGGATCAGGTAGAT |
| NiV bait-28 | TTGCCTATCCCAGAATATACTGAAGTGGATAACAATCATCTTATATATGATCCAGACCCCGTTTCAGAAATAGATTGCAGCCGTCTTTCTAATCAGGAGTCCAAATCAAGAGAATTAGAC |
| NiV bait-29 | CACAGGGTCCTTACCTTCATCAACAAGATTTTGAAAAGCTGTCTCAAAATCTCCTTGTAACATCTTACATGATTTATCTAATGAACTGGTGTGACTTCAAGAAATACCCCTTTTTAATCG |
| NiV bait-30 | GGAGCTCAAAGGTTGTTTATAGGAGAAGGTTCTGGGAGCATGATGTTATTATATCAGTCTACATTGGGGCAATCAATTTCTTTTTACAATTCAGGTATAGATGGAGATTATATACCAGGT |
| NiV bait-31 | CAGTTCCATGAAGATCTAAAGAAATACTATCATATTGACCAACCTTTCTTTGTACCAACTAAAATCACTAGTGATGAACAAGTACTTCTCCAAGCAGGGCTAAAACTCAATGGGCCAGAA |
| Henipa bait-01 | TCGAAGCCCAAATTTGGATCCTGATTGCAAAGGCAGTGACTGCTCCAGATACAGCCGAGGAAAGTGAGACCAGAAGATGGGCAAAGTATGTTCAACAAAAGAGGGTCAATCCATTCTTTG |
| Henipa bait-02 | AGTCTAAAGGAGAAAGAAACTAAACAAGCAGGAAGATTATTTGCTAAAATGACATATAAGATGCGTGCCTGTCAGGTAATTGCTGAAGCTTTGATTGCATCGGGAGTGGGCAAGTATTTT |
| HeV bait-01 | ACTTAGGAACCAAGACAGTGACAATTGGTCTTGGTATTGGACAATTGTTCAAGGTTCCAAAATGAGTGATATATTTGAAGAGGCGGCTAGTTTCAGAAGCTATCAATCGAAACTCGGTCG |
| HeV bait-02 | GATATGAGCACTCTTGTGTCAGCCGTCATTACCATCGAAGCCCAAATTTGGATCCTGATTGCAAAGGCAGTGACTGCTCCAGATACAGCCGAGGAAAGTGAGACCAGAAGATGGGCAAAG |
| HeV bait-03 | AAATCAGCAAGACATCATGCTGGCGGGATTGATCAAAACATGGCTAATAAACTTGGATTAAATTCTGATCAAGTTGCAGAACTGGCTGCTGCAGTTCAAGAAACATCAGTTGGGAGACAG |
| HeV bait-04 | CGTGTGACGCTTAATCAGAAACTCATATAAGCTCCAAATCATTCTCTATGACACACTCTTATAAATAAATTTAAGAGAGTGGATTGAGATTGAGTTATGGACCTGTGTCATAATATACCT |
| HeV bait-05 | AAATGGACAAGTTGGATCTAGTCAATGATGGCCTCGATATTATTGACTTTATTCAGAAGAACCAAAAAGAAATACAAAAGACATACGGACGATCAAGCATCCAACAACCAAGTACCAAAG |
| HeV bait-06 | AGATCTCTCAAAATCTCCTCCTAGAGGTAATGTCAATCTGGACAGCATCAAGATTTACACTTCGGACGACGAGGATGAAAATCAGCTGGAGTATGAGGATGAGTTTGCCAAAAGCTCAAG |
| HeV bait-07 | CGTCCAGAGACCAGGCACACCGATGCCTAAATCAAGGATCATGCCCATTAAAAAGGGCACAGACGCGAAGTCTCAATATGTTGGGACGGAAGACGTGCCTGGGTCGAAGAGTGGTGCAAC |
| HeV bait-08 | AGTGTTGGCTAAGACCAATACTGCATTGTCGACAATAGAAGGACACCTAGTCTCGATGATGATCATGATACCAGGGAAAGGCAAGGGTGAAAGGAAAGGGAAAACAAATCCGGAGCTAAA |
| HeV bait-09 | AGTAGCAGTCAGACCAAGATCATATATTAGAGGTACACATCATAATGGCCAGTGTCTTAATCTCAACCATTCATAAACCGTCAGTGTTTCCCTTATAAACTCCATTATGTAGTTTATTAA |
| HeV bait-10 | CTTGGACTTAAAGAGAGACAATCATTCAGCCCCGTCCAAGACACACCTGAGTGGCCAGAACCATGGATTTTAGTGTGAGTGATAACCTTGATGATCCAATAGAAGGTGTTTCAGATTTTA |
| HeV bait-11 | TTCAATCTTCTAGTATACCTCAAAATCGATGCAGATCTTGCAAAAGCTGGAATCCAAGGAAGCTTCGACAAAGACGGAACCAAAGTGGCATCTTTCATGCTCCATCTCGGGAATTTTGTC |
| HeV bait-12 | TGACAACACAGGGAAGATCCTAAAGGGGTGAAATAAAACATTCACTTGGTTATTGTAGATTGATAAATTAACCAGATAACCACTTTAATCTTCATTCTAAAGAATGCCACAGTGTCAGTA |
| HeV bait-13 | TATCTTGGCAAACACTACACTAACCTCGTCTGTACTGTCTGAAAGTGGTCAGGATTGATTGACAGTTATTATTTATCTTTAAGCAATGGCTACACAAGAGGTCAGGCTAAAGTGTTTGCT |
| HeV bait-14 | GAACGCAGACAATATCAATAAACTCAAGAGCAGCATAGAGTCTACAAATGAGGCTGTTGTCAAATTACAGGAAACAGCTGAGAAAACAGTCTACGTCCTTACTGCTCTTCAAGATTACAT |
| HeV bait-15 | GACTATGCTACACCCATGACGGCTAGCGTGAGAGAATGCTTGACAGGATCCACAGATAAGTGCCCAAGGGAGTTAGTAGTCTCATCCCATGTTCCAAGATTTGCCCTCTCAGGAGGAGTC |
| HeV bait-16 | TGATCACTTTCATAAGCTTTGTAATAGTTGAGAAAAAGAGAGGGAATTACAGCAGGCTAGATGATAGGCAAGTGCGACCGGTCAGTAATGGTGATCTGTATTATATTGGAACATAAAATA |
| HeV bait-17 | TTACTGTGATGGATAAAGGGACTTTACAAAAAACTTAGGACCCAAGTCCTTAACCACATTCTAATGTGAGGGAGAATTAAATGTACATTGAGACTGACAATCTAATATACAGAGTGTTTG |

Table S2. Hybridization probes set-2 which is a combination of set-1 and HeV-g2 sequences

| Henipa Bait-01 | GGGGAAATATGGATACGTGTTAAAAAACTGCTTATGTCTAAAACTTAGGAACCAAGACAGTGATAATTGGTCTTGGTATTGGATAACTATTCAAGGTGTCGAAATGGGTGATATATTTGA |
| --- | --- |
| Henipa Bait-02 | TTGGTCTTGGTATTGGATAACTATTCAAGGTGTCGAAATGGGTGATATATTTGAAGAAGCGGCCAGTTTCAGAAACTACCAATCGAAACTCGGTCGAGATGGGCGGGCAAGTGCAGCAAC |
| Henipa Bait-03 | TTTCAGAAACTACCAATCGAAACTCGGTCGAGATGGGCGGGCAAGTGCAGCAACAGCTACTTTGACTACTAAGATAAGAATTTTTGTACCAGCGACTAATAGTCCAGAGCTGAGATGGG |
| Henipa Bait-04 | TCATCATCGATGTGGGGTCTATGCTCAACGGGATTCCTGTGATGGAACGAAGAGGGGACAAGGCACAAGAAGAAATGGAAGGTCTGATGAGGATTTTGAAAACTGCACGTGAGAGCAGCA |
| Henipa Bait-05 | GTGAGAGCAGCAAGGGGAAAACCCCCTTTGTTGATAGCAGAGCCTACGGAATGAGGATCACAGATATGAGCACCCTTGTATCAGCTGTCATTACTATTGAAGCTCAAATTTGGATCTTGA |
| Henipa Bait-06 | TTTGGATCTTGATTGCTAAAGCAGTGACTGCTCCGGACACGGCCGAGGAAAGTGAGACCAGGAGATGGGCTAAGTATGTTCAACAGAAAAGAGTCAATCCATTCTTTGCTTTGACCCAAC |
| Henipa Bait-07 | CTTTGACCCAACAATGGCTGACAGAAATGAGGAATCTTCTCTCGCAGAGTCTCTCGGTGAGAAAATTCATGGTGGAAATCTTGATGGAAGTCAAGAAAGGCGGATCAGCAAAAGGAAGGG |
| Henipa Bait-08 | CAAAAGGAAGGGCTGTCGAGATAATATCTGATATAGGAAATTATGTTGAAGAAACGGGAATGGCTGGCTTCTTTGCAACGATCAGATTTGGTCTTGAAACGAGATACCCTGCGCTTGCAC |
| Henipa Bait-09 | CTGCGCTTGCACTCAATGAGTTCCAGAGCGATCTTAATACTATCAAAGGACTGATGCTGCTTTACAGAGAAATAGGACCTCGAGCACCATATATGGTGCTTCTTGAGGAATCTATTCAGA |
| Henipa Bait-10 | AATCTATTCAGACAAAGTTCGCGCCTGGCGGTTACCCACTTCTGTGGAGTTTTGCTATGGGTGTTGCAACTACAATTGACCGATCTATGGGTGCCCTCAACATCAATCGCGGTTATCTTG |
| Henipa Bait-11 | GCGGTTATCTTGAACCTATGTACTTCAGGCTCGGACAGAAATCAGCGAGACATCATGCTGGCGGGATCGACCAGAACATGGCAAATAAACTTGGACTAAATCCTGATCAAGTTGCAGAAC |
| Henipa Bait-12 | AAGTTGCAGAACTGGCTGCTGCAGTTCAGGAAACATCAGTTGGAAGGCAGGATAACAACATGCAGGCAAGGGAGGCTAAGTTTGCAGCTGGAGGAGTACTTGTTGGAGGTGGTGAGCAAG |
| Henipa Bait-13 | GTGGTGAGCAAGACATTGATGAGGATGAGGAACCAATCGAACACAGTGGAAGGCAATCTGTCACTTTCAAAAGAGAAATGAGCATGTCATCCCTTGCTGACAGTCTCCCGAGCAGTTCAG |
| Henipa Bait-14 | AACACCTAGGAGTAATACAAATCTATCATACTAGGTAACAATATATAATTAGTATAGGCCTGTGTGACTTGATTGGTATATTAAGAAAAACTTAGGATCCAAGACTATAAATCTAGGATC |
| Henipa Bait-15 | TAAATCTAGGATCTCTTGCAATCTTGACCCTTGGCAAAAGACCATATACAAAGTTCAATTCTCTTAATCAAACGGTATTTCTGACTAATAAATGGACAAGCTGGATTTAGTTAATGATGG |
| Henipa Bait-16 | TAGTTAATGATGGCCTCAATATTATTGACTTTATTCAAAAGAACCAAAAAGAAATACAAAAGACATACGGACGATCAAGCATCCAACAACCAAGTACCAAAGACAGGACAAGAGCATGGG |
| Henipa Bait-17 | ACAAGAGCATGGGAGGACTTCTTGCAGAGCACCGGTGGAGAACATGAACAGGCTGAGGGGAGAGTGTCTAAGAATGATGGAAGTACTGAAGGAAGAGGTGTGGAGGATATACCCAGTACT |
| Henipa Bait-18 | TATACCCAGTACTGCTTCCTCAGATGGAACTATTGGACAAAGAGTGTCAAACACCCGAGATTGGGCAGAGGGTTCAGATGATATACAACTGGACCCAATGGTTACAGACGTTATATACCA |
| Henipa Bait-19 | GAAGAGCCTCTGGTAGTCCCAGAATATTACTACGGGACTGGAAGGAAGGGAGATCTATCAACATCTTCTCCTAAAGGTAATGTCAATTTGGGCAGCATCAGGATGTACACTTCAGATGAC |
| Henipa Bait-20 | TACACTTCAGATGACGAAGATGACAATCAGCTGGAGTATGAGGATGAGTTTGCCAAAAGCTCAAGTGAAGTCATTATTGACACTACTCCTGAGGACAATGATTCCATCAATCAGGAAGAA |
| Henipa Bait-21 | ATAATACAAGAACTAGAACGGGAAGGTTCTCATCCAGGAGATCCATTATGTTTGAAAGAATCACCTCAGCTGTCAGGAAATTCCAGGGGTCAACTTGATCGCCAGCTGAACACAAACGAT |
| Henipa Bait-22 | ACACAAACGATTCAACACCTTTTGGAGGTGTCCAAAGACCAGGCACACCAATGCCCAAGTCCAAGCCCATGCCCATTAAAAGGGGCACAGGCGCGAAATCTCAGTATGTTGGGATGGAAG |
| Henipa Bait-23 | TGGGATGGAAGACGTGCCTGGGTCGAAGAGTGGTGCAACCCGGCATGTTCACGGATCACCCTCCAACCAAGAAGGCAAGAATGTCACTGCGGAGAATGTCCAACTGAGTGCTCTCAGTGC |
| Henipa Bait-24 | GCTCTCAGTGCTGCCAAGACGAGTGAAGGGCAAGATCTAGATGCCACAGATAATGATGATAGCCTAGATGACAAGTATATCATGCCATCAGATGATTTTGCTAACACTTTCTTACCTCAC |
| Henipa Bait-25 | TCTTACCTCACGACACCGATAGGTTAAACTATCATGCAGACCACCTAAATGACTACGATTTAGAGACCTTGTGTGAAGAATCAGTATTGATGGGAGTCATCAATGCAATTAAACTTATTA |
| Henipa Bait-26 | TAAACTTATTAATATTGATATGAGGTTAAACCACATTGAGGACCAGATGAAAGAAATCCCCAAGATTATTAATAAGATAGATTCTATTGACCGGGTGTTAGCTAAGACTAATACTGCATT |
| Henipa Bait-27 | AATACTGCATTGTCAACAATAGAAGGACACTTAGTTTCAATGATGATCATGATACCAGGGAAAGGCAAGGGTGAAAGGAAAGGGAAAAATAATCCAGAGTTAAGACCTGTGATCGGGAGG |
| Henipa Bait-28 | TGATCGGGAGGAATGTCTTGGAACAACAAGAATTATTCTCATTTGATAACCTCAAAAACTTCAGAGACGGTTCATTGACCGACGAACCTTATGGAGGGGCAGCCCGAATGAGAGATGATC |
| Henipa Bait-29 | GAGAGATGATCTGATTTTGCCCGAACTTAATTTCAGTGAGACAAATGCATCACAGTTCATTCCTTTGGCAGATGATGCATCCAAGGATGTCGTGAGGACTATGATTAGGACTCACATCA |
| Henipa Bait-30 | CTGGGAAACGCAAGAAAATCAGGACAATTGCTGCATACCCTCTTGGTGTTGGTAAGAGTACTTCTCACCCCCAAGACCTTTTAGAAGAACTATGCTCTTTGAAGGTCACAGTCAGGAGAA |
| Henipa Bait-31 | ACAGTCAGGAGAACAGCTGGTGCTACAGAGAAAGTTGTGTTTGGATCCTCAGGCCCCCTTCACCATCTCTTACCATGGAAAAAAGTTTTGACTGGCGGGTCAATATTCAATGCTGTCAAG |
| Henipa Bait-32 | CAATGCTGTCAAGGTTTGCCGTAACGTAGATCAGATTCAACTGGAAAAGCAACAATCATTGAGGATTTTTTTCCTAAGTATTACCAAATTGAATGATTCCGGTATCTACATGATCCCAAG |
| Henipa Bait-33 | ACATGATCCCAAGAACAATGTTGGAATTCAGAAGGAACAATGCCATTGCTTTTAATCTTCTGATATACCTCAAAATTGATGCAGATCTTGCAAAAGCCGGAATCCAAGGGAGTCTCGACA |
| Henipa Bait-34 | GGGAGTCTCGACAAGGACGGAGCTAAAGTGGCATCCTTCATGCTTCACCTTGGTAATTTTGTTCGACGAGCCGGGAAGTATTACTCCGTTGAATACTGCAAGAGAAAGATCGACAGAATG |
| Henipa Bait-35 | GATCGACAGAATGAAGCTTCATTTCTCTCTCGGTTCAATTGGCGGTTTAAGTCTACACATCAAAATCAATGGGGTGATTAGCAAGAGATTATTTGCCCAGATGGGTTGCCAGAAGAAC |
| Henipa Bait-36 | CAAGATGATACCTAATGTTTCAAATGTCTCAAAATGCACCGGAACTGTTATGGAGAATTACAAAAACAGACTTACAGGGATTCTCTCCCCAATTAAGGGTGCTATTGAGCTGTACAATAA |
| Henipa Bait-37 | CCCAATTAAGGGTGCTATTGAGCTGTACAATAACAACACACATGACTTAATTGGTGATGTTAAGCTTGCTGGTGTAGTGATGGCAGGGATTGCAATCGGGATAGCTACTGCCGCACAAAT |
| Henipa Bait-38 | GATTGCAATCGGGATAGCTACTGCCGCACAAATCACAGCAGGGGTGGCCTTGTATGAGGCAATGAAGAATGCAGACAACATCAATAAGCTCAAGAGCAGCATAGAATCCACAAACGAAGC |
| Henipa Bait-39 | GCTCAAGAGCAGCATAGAATCCACAAACGAAGCTGTTGTCAAATTGCAAGAGACAGCTGAAAAAACAGTCTACGTTCTTACTGCTCTTCAGGACTACATTAACACAAACCTTGTCCCCAC |
| Henipa Bait-40 | TTACTTAGAACACTTGGTTATGCGACAGAAGATTTCGACGACCTTCTAGAAAGTGACAGCATAACAGGTCAGATAGTTTATGTAGATCTCAGCAGCTATTATATAATAGTAAGGGTATAT |
| Henipa Bait-41 | TATATAATAGTAAGGGTATATTTCCCTATACTAACTGAAATCCAACAAGCTTATGTGCAGGAGTTACTCCCAGTGAGCTTCAACAATGACAACTCAGAATGGATTAGCATTGTCCCAAAT |
| Henipa Bait-42 | TGGATTAGCATTGTCCCAAATTTCGTGCTGATTAGGAACACACTGATCTCAAATATAGAGGTCAAGTACTGCTTAACTACCAAGAAGAGCGTGATCTGTAATCAAGATTACGCTACACCC |
| Henipa Bait-43 | AATCAAGATTACGCTACACCCATGACATCTACTGTGAGAGAATGCTTGACGGGATCTACAGATAAATGTCCAAGAGAATTAGTAGTTTCATCCCATGTTCCAAGGTTTGCTCTCTCAGGA |
| Henipa Bait-44 | CCAAGGTTTGCTCTCTCAGGAGGAGTCTTGTTTGCAAATTGCATAAGCGTGACATGTCAATGTCAGACTACCGGCAGGGCAATATCTCAATCAGGGGAACAGACATTACTGATGATTGAC |
| Henipa Bait-45 | CTGGGATCTAAGATAAGTCAGTCTACCAGTAGCATCAATGAGAATGTCAATGATAAATGCAAATTTACTCTCCCTCCTCTTAAAATTCATGAATGTAATATCTCTTGTCCGAATCCTCTG |
| Henipa Bait-46 | CCTCTGCCTTTCAGAGAATACCGACCAATTTCACAAGGAGTAAGTGATCTTGTAGGATTGCCGAACCAAATCTGTTTACAAAGGACAACATCAACAATCTTGAAGCCCAAGTTAATATCC |
| Henipa Bait-47 | ATATCCTATACCCTGCCAATCAATACCAGGGAAGGGGTTTGCATCACTGATCCGCTCTTGACTATTGATAATGGTTTCTTTGCCTATAGTCATCTCGAGAAGATTGGATCATGTACTAGA |
| Henipa Bait-48 | ATAATGAATCTCCATGTCGAATAAATATCATGTCAGCTTTAATCGATAGGAACCAGTCAGACCAAAAAAGACATCACATTCACCCAAATACGCGAAATCGCCACAAATGCAATAACACAA |
| Henipa Bait-49 | AAAAAGACATCACATTCACCCAAATACGCGAAATCGCCACAAATGCAATAACACAAGTAAGACATTCCTTGATTATCATACAGAGTTTAGTCCTTACAGTCCAGACAGAGTGGATAGGAC |
| Henipa Bait-50 | TTCCTTGATTATCATACAGAGTTTAGTCCTTACAGTCCAGACAGAGTGGATAGGACAGAAGCATCTGATTTCTCGAAATATGATGATGGAACAGGAACAAAATTTGACACAGTGAGTGC |
| Henipa Bait-51 | TGATGAGATTTATGGCTTACCTGGTTTTTTCAATTGGATGCATAAACGATTGGAGAAGTCAGTTATTTATGTTGCAGATCCTAATTGTCCACCGGACATTGGAAAACACATGAATCTTGA |
| Henipa Bait-52 | ATTTATGTTGCAGATCCTAATTGTCCACCGGACATTGGAAAACACATGAATCTTGATGATACTCCTGAGGACGACATATTTATCCACTCACCAAAGGGCGGGATTGAGGGTTATAGTCAG |
| Henipa Bait-53 | CTGAGGACGACATATTTATCCACTCACCAAAGGGCGGGATTGAGGGTTATAGTCAGAAGACTTGGACAATAGCAACGATTCCTTTTCTTTTTCTCAGTGCTTATGAGACAAATACAAGAA |
| Henipa Bait-54 | GAAAGTCATACAGCAACTGCTTATCTCAACTGAGTTTAGTATTAATGAGACATTGACTGTTGATGTAACATCCCCGATATCCAATAATCTAGACTGGCTGGTAACTGCATCTTTAATTCC |
| Henipa Bait-55 | TCTTTAATTCCTGCACCAATTGGGGGCTTCAATTACTTGAATCTATCAAGAATATTTGTAAGAAACATAGGAGACCCTGTGACTGCATCACTAGCAGATCTCAAAAGAATGATAGAACAT |
| Henipa Bait-56 | TGATAGAACATAGCTTGATGACAGATAAAGTCCTCCAAAAAGTAATGAACCAAGAACCAGGTGACGCAAGTTTTCTAGATTGGGCCAGTGACCCATACTCAGGGAATTTACCGGATTCTC |
| Henipa Bait-57 | ACCGGATTCTCAGAGCATCACAAAAACAATCAAGAACATAACAGCTAGAACTATATTGAGGACATCACCAAACCCTATGTTGAAGGGTCTGTTCCATGATAAATCATTTGAAGAAGATCT |

Table S3: Details on the flying foxes tested in this study, including year and month of collection, species, location, ABLV and HeV-g2 results (positive results are highlighted in grey) and the suspected cause of death. GHFF: grey headed flying fox; BFF: black flying fox; LRFF: little red flying fox; UNSP FF: unspecified flying fox.

| **Year** | **Month** | **Species** | **Location** | **State** | **ABLV** | **HeV-g2** | **Cause of death** |
| --- | --- | --- | --- | --- | --- | --- | --- |
| 2013 | Jan | GHFF | Adelaide | SA | neg | pos | suspect heat stress event |
| 2014 | Jan | BFF | unspecified | WA | neg | neg | no history provided |
| 2014 | Apr | GHFF | Melbourne | VIC | neg | neg | no history provided |
| 2014 | Apr | GHFF | Melbourne | VIC | neg | neg | no history provided |
| 2014 | May | GHFF | Melbourne | VIC | neg | neg | no history provided |
| 2014 | May | UNSP FF | Bribie Island | QLD | neg | neg | contact with dog |
| 2014 | May | GHFF | Melbourne | VIC | neg | neg | no history provided |
| 2014 | May | GHFF | Adelaide | SA | neg | neg | no history provided |
| 2014 | May | GHFF | Melbourne | VIC | neg | neg | hit by car |
| 2014 | Jun | GHFF | Melbourne | VIC | neg | neg | no history provided |
| 2014 | Jun | GHFF | Adelaide | SA | neg | neg | no history provided |
| 2014 | Jun | GHFF | Melbourne | VIC | neg | neg | caught in fence |
| 2014 | Jul | GHFF | Melbourne | VIC | neg | neg | bitten by dog |
| 2014 | Sep | GHFF | Melbourne | VIC | neg | neg | bitten by dog |
| 2015 | Jan | GHFF | Adelaide | SA | neg | neg | contact with human |
| 2015 | Feb | GHFF | Bairnsdale | VIC | neg | neg | contact with human |
| 2015 | Dec | LRFF | Broome | WA | neg | pos | bitten by dog |
| 2016 | May | LRFF | Perth | WA | neg | neg | no history provided |
| 2016 | Jun | BFF | Broome | WA | neg | neg | bitten by dog |
| 2017 | Jan | GHFF | Melbourne | VIC | neg | neg | caught in fruit netting |
| 2017 | Jan | LRFF | Broome | WA | neg | neg | unknown |
| 2017 | Jan | GHFF | Melbourne | VIC | neg | neg | contact with human |
| 2017 | Feb | GHFF | Melbourne | VIC | neg | neg | caught in fruit netting |
| 2017 | Feb | GHFF | Melbourne | VIC | neg | neg | bitten by dog |
| 2017 | Aug | UNSP FF | Broome | WA | neg | neg | bitten by cat |
| 2017 | Dec | GHFF | Melbourne | VIC | neg | neg | unspecified trauma |
| 2018 | Feb | GHFF | Melbourne | VIC | neg | neg | unspecified trauma |
| 2018 | Feb | UNSP FF | Melbourne | VIC | neg | neg | bitten by dog |
| 2018 | Apr | GHFF | Melbourne | VIC | pos | neg | ABLV |
| 2018 | Apr | GHFF | Melbourne | VIC | neg | neg | unspecified trauma |
| 2018 | May | GHFF | Melbourne | VIC | neg | neg | caught in fruit netting |
| 2018 | Apr | GHFF | Melbourne | VIC | neg | neg | no history provided |
| 2018 | May | GHFF | Melbourne | VIC | neg | neg | unspecified trauma |
| 2018 | Mar | GHFF | Melbourne | VIC | neg | neg | caught in fruit netting |
| 2018 | Mar | GHFF | Melbourne | VIC | neg | neg | caught in fruit netting |
| 2018 | Jun | GHFF | Melbourne | VIC | neg | neg | unknown |
| 2019 | Jan | GHFF | Melbourne | VIC | neg | neg | unspecified trauma |
| 2019 | Jan | GHFF | Bairnsdale | VIC | neg | neg | suspect heat stress event |
| 2019 | Feb | UNSP FF | Burleigh Heads | QLD | neg | neg | bitten by dog |
| 2019 | Feb | GHFF | Wollongong | NSW | neg | neg | contact with human |
| 2019 | Feb | GHFF | Melbourne | VIC | neg | neg | contact with human |
| 2019 | Feb | GHFF | Melbourne | VIC | neg | pos | caught in fruit netting |
| 2019 | Feb | GHFF | Melbourne | VIC | neg | neg | caught in fruit netting |
| 2019 | Feb | UNSP FF | Brisbane | QLD | neg | neg | no history provided |
| 2019 | Mar | GHFF | Melbourne | VIC | neg | neg | caught in fruit netting |
| 2019 | Mar | GHFF | Melbourne | VIC | neg | neg | caught in barbed wire fence |
| 2019 | Mar | GHFF | Melbourne | VIC | neg | neg | electrocuted |
| 2019 | Mar | UNSP FF | Melbourne | VIC | neg | neg | shot |
| 2019 | Mar | GHFF | Melbourne | VIC | neg | pos | caught in fruit netting |
| 2019 | Mar | GHFF | Melbourne | VIC | neg | neg | unspecified trauma |
| 2019 | Mar | GHFF | Melbourne | VIC | neg | neg | cranial trauma |
| 2019 | Apr | UNSP FF | Melbourne | VIC | neg | neg | bitten by dog |
| 2019 | Mar | GHFF | Melbourne | VIC | neg | neg | bitten by dog |
| 2019 | Apr | GHFF | Melbourne | VIC | neg | neg | bitten by dog |
| 2019 | Mar | GHFF | Melbourne | VIC | neg | neg | no history provided |
| 2019 | Apr | UNSP FF | Bairnsdale | VIC | neg | neg | contact with human |
| 2019 | Apr | GHFF | Melbourne | VIC | neg | neg | bitten by dog |
| 2019 | Apr | GHFF | Melbourne | VIC | neg | neg | no history provided |
| 2019 | Apr | GHFF | Melbourne | VIC | neg | neg | caught in fruit netting |
| 2019 | May | GHFF | Melbourne | VIC | neg | neg | unspecified trauma |
| 2019 | May | UNSP FF | Tallygaroopna | VIC | neg | neg | bitten by dog |
| 2019 | Jun | BFF | Broome | WA | neg | neg | unknown |
| 2019 | Jun | UNSP FF | Broome | WA | neg | neg | contact with human |
| 2019 | Jul | GHFF | Melbourne | VIC | neg | neg | no history provided |
| 2019 | Nov | GHFF | Melbourne | VIC | neg | neg | no history provided |
| 2020 | Jan | GHFF | Melbourne | VIC | neg | neg | contact with human |
| 2020 | Jan | GHFF | Melbourne | VIC | neg | neg | no history provided |
| 2020 | Jan | GHFF | Melbourne | VIC | neg | pos | no history provided |
| 2020 | Jan | GHFF | Melbourne | VIC | neg | pos | trauma unspecified |
| 2020 | Jan | GHFF | Melbourne | VIC | neg | neg | no history provided |
| 2020 | Jan | GHFF | Melbourne | VIC | neg | pos | bitten by dog |
| 2020 | Jan | GHFF | Melbourne | VIC | neg | neg | caught in fruit netting |
| 2020 | Feb | GHFF | Melbourne | VIC | neg | neg | suspect heat stress event |
| 2020 | Feb | GHFF | Mangerton | NSW | neg | neg | abandoned baby |
| 2020 | Feb | GHFF | Melbourne | VIC | neg | neg | caught in barbed wire fence |
| 2020 | Feb | GHFF | Melbourne | VIC | neg | neg | fractured wing |
| 2020 | Feb | GHFF | Bairnsdale | VIC | neg | neg | fractured wing |
| 2020 | Mar | GHFF | Melbourne | VIC | neg | neg | fractured wing |
| 2020 | Mar | GHFF | Yagoona | NSW | neg | neg | neurological signs |
| 2020 | Mar | GHFF | Melbourne | VIC | neg | neg | contact with dog |
| 2020 | Mar | GHFF | Melbourne | VIC | neg | neg | electrocuted |
| 2020 | Mar | UNSP FF | Kangaroo Flat | VIC | neg | neg | bitten by dog |
| 2021 | Jan | GHFF | Melbourne | VIC | neg | neg | caught in fruit netting |
| 2021 | Jan | GHFF | Gippsland | VIC | neg | neg | injuries from tree collapse |
| 2021 | Jan | GHFF | Gippsland | VIC | neg | neg | injuries from tree collapse |
| 2021 | Feb | GHFF | Melbourne | VIC | neg | neg | caught in barbed wire fence |
| 2021 | Jan | GHFF | Sale | VIC | neg | neg | orphaned from heat stress event |
| 2021 | Feb | GHFF | Melbourne | VIC | neg | pos | fractured wing |
| 2021 | Feb | GHFF | Adelaide | SA | neg | pos | dog attack |
| 2021 | Feb | GHFF | Melbourne | VIC | neg | neg | suppurative peritonitis |
| 2021 | Apr | GHFF | Kangaroo Flat | VIC | neg | neg | contact with human |
| 2021 | Mar | GHFF | Adelaide | SA | neg | pos | dog attack |
| 2021 | Mar | GHFF | Melbourne | VIC | pos | pos | ABLV |
| 2021 | Mar | GHFF | Adelaide | SA | neg | neg | contact with human |
| 2021 | Mar | GHFF | Melbourne | VIC | neg | neg | unknown |
| 2021 | Mar | GHFF | Melbourne | VIC | neg | neg | caught in fruit netting |
| 2021 | Mar | GHFF | Melbourne | VIC | neg | neg | cervical fracture |
| 2021 | Mar | GHFF | Adelaide | VIC | pos | neg | ABLV |
